# Increased oxidative stress, inflammation and fibrosis in perirenal adipose tissue of patients with cortisol-producing adenoma

**DOI:** 10.1080/21623945.2019.1690834

**Published:** 2019-11-13

**Authors:** Chunyan Wu, Huijian Zhang, Jiajun Zhang, Hongbin Zhang, Yanmei Zeng, Shu Fang, Ping Li, Yudan Zhang, Xiaochun Lin, Ling Wang, Yaoming Xue, Meiping Guan

**Affiliations:** a Department of Endocrinology and Metabolism, Nanfang Hospital, Southern Medical University, Guangzhou, China; b Department of Urology, Nanfang Hospital, Southern Medical University, Guangzhou, China; c Department of Radiology, National Cancer Center/National Clinical Research Center for Cancer/Cancer Hospital & Shenzhen Hospital，Chinese Academy of Medical Sciences and Peking Union Medical College, Shenzhen, China; d Department of Biomedical Sciences, University of Copenhagen, Copenhagen, Denmark

**Keywords:** Cushing’s syndrome, oxidative stress, inflammation, fibrosis, adipose tissue, microarray

## Abstract

Although much is known about that corticosteroids affect the functions of adipose tissues, little genetic information is available for perirenal adipose tissue (peri-N) from patients with cortisol-producing adenoma (CPA). We conducted microarray analysis of peri-N from patients with CPA by using an Affymetrix human U133 plus 2.0 array. We also analysed the inflammation, fibrosis and oxidative stress in vitro. Compared with normotension (NT) group, CPA group has significantly higher protein levels of TNFα, IL-6, fibronectin (FN) and collagen I (COLI). The protein level of NADPH oxidase 4 (Nox4) significantly increased, while nuclear factor erythroid 2-related factor 2 (Nrf2) and hemeoxygenase-1 (HO-1) levels were significantly reduced in the CPA group. Dexamethasone markedly induced fibrosis and adipogenesis-related gene expression in predifferentiated stromal vascular fraction (SVF) cells, 3T3-L1 preadipocytes and brown preadipocytes. Chronic exposure to endogenous glucocorticoids due to CPA increases peri-N oxidative stress, inflammation and fibrosis, which may contribute to the metabolic disturbances associated with hypercortisolism in these patients.

## Introduction

Patients with Cushing’s syndrome (CS) often suffer from a series of complications such as impaired glucose tolerance, dyslipidemia and cardiovascular disease (CVD) [1,2]. A previous study showed that the mortality in patients with CS still increased despite the aetiology treatment [3]. However, the relevant mechanisms remain unclear. A recent study showed that perirenal adipose tissue (peri-N) from patients with active Cushing’s disease exhibits an increased number of pro-inflammatory macrophages compared to body mass index (BMI) matched controls [4]. Whether endogenous hypercortisolism can lead to peri-N dysfunction in patients with CPA remains unclear. Peri-N was showed brown adipose tissue (BAT) characterization [5]. Our previous study [6] reported that high aldosterone levels in patients with primary aldosteronism due to adrenal adenoma induced peri-N inflammation and fibrosis and let to dysfunction. A comprehensive assessment of perirenal fat from patients with CPA is still lacking. Very little genetic information about peri-N in CPA is available. In the current study, RNA sequencing of perirenal fat in CPA patients was performed by using gene microarray. Adipose tissue oxidative stress can promote adipose tissue inflammation and inappropriate extracellular matrix remodelling in obesity [7], however, data of peri-N characteristics from patients with CPA are lacking. We also examined oxidative stress, inflammation and fibrosis in peri-N from CPA patients, SVF cells, 3T3-L1 and brown preadipocytes.

## Patients and methods

### Patients

Peri-N and sub-Q were sampled from 8 patients with benign CPA undergoing laparoscopic adrenalectomy, 4 patients with essential hypertension (EH) and 10 patients with NT undergoing nephrolithotomy or ureterolithotomy. The diagnosis of Cushing’s syndrome was made according to guidelines [8]. CPA was diagnosed according to clinical characteristics, biochemical assessment, adrenal glands computed tomography scan and histopathology. Serum cortisol, 24h urinary free cortisol and adrenocorticotropic hormone were used for initial screening. The 1mg dexamethasone suppression test (DST) and 8mg high-dose DST were used for subsequent diagnosis of CPA. All CPA patients were in active disease and did not receive reduce hypercortisolemia treatment before surgery. The ethics committee of Nanfang Hospital approved this study. .All individual participants provided written informed consent.

### Gene expression profile of peri-N in patients with CPA and EH

Gene expression in peri-N of 5 patients with CPA and 4 patients with EH was measured by using Affymetrix HG U133 plus 2.0 arrays. The characteristics of the patients included in the gene expression analyses are showed in Table 1.

**Table 1.** Clinical and biochemical feature of the patients.

Parameter	Essential hypertension	Cushing’s syndrome	*P* value
n	4	5	
Sex(M/F)	4/0	0/5	
Age(year)	54.25 ± 7.97	36.80 ± 14.52	NS
Body mass index(kg/m^2^)	24.59 ± 2.99	26.33 ± 5.36	NS
Systolic blood pressure(mmHg)	143.50 ± 17.25	131.80 ± 14.58	NS
Diastolic blood pressure(mmHg)	89.50 ± 5.45	95.40 ± 9.29	NS
Fasting glucose(mmol/L)	5.25 ± 0.40	5.84 ± 1.76	NS
White blood cell count (10^9^/L)	7.44 ± 1.55	9.55 ± 1.67	NS
Alanine transaminase (U/L)	25.00 ± 9.055	31.40 ± 18.03	NS
Glutamic-oxal(o)acetic transaminase (U/L)	24.5 ± 4.04	24.4 ± 16.04	NS
Morning plasma cortisol levels (µg/dl)		23.08 ± 6.89	
(normal value, 4.3–22.4)			
Midnight plasma cortisol levels (µg/dl)		22.59 ± 3.08	
(normal value, 3.9–16.66)			

All data are shown as mean ± Standard Deviation. NS, Not significant.

### Quantitative real-time PCR and western blotting

Peri-N and sub-Q from patients with CPA (n = 8) and patients with NT (n = 10) were used for validation experiments. The characteristics of the patients included in the validation experiment are described in Table 2. Adipose tissue total RNA was extracted by using Trizol (TAKARA) and used for cDNA synthesis using Prime Script RT Reagent Kit (TAKARA). The quantitative real-time PCR (qRT-PCR) was run on Roche LightCycler 480 Real-Time PCR System. Gene expression levels of inflammation, fibrosis, oxidative stress and adipogenesis marker genes was analysed by qRT-PCR. Arbp (38B4) served as housekeeping gene in mouse brown preadipocytes and 3T3-L1 preadipocytes. 18S were used as housekeeping gene in human SVF cells and adipose tissue. Gene expression levels of inflammation, fibrosis, oxidative stress and adipogenesis marker genes were measured by qRT-PCR. Western blot was carried out as previously described [9]. For the analysis of total protein in peri-N, antibodies specific for interleukin-6 (Cell Signalling Technology, #12,153), HO-1 (Abclonal, A1346), NOX4 (Abclonal, A11274) and Nrf2 (Santa Cruz, sc-722) were used. For the analysis of nuclear protein in perirenal adipose tissue, antibodies for phospho-NF-κB p65 (Cell Signalling Technology, #3033) and NF-κB p65 (Cell Signalling Technology, #8242) were used. β-acitn and histone-H3 were used as total protein and nuclear protein loading controls, respectively.

**Table 2.** Clinical and biochemical feature of the patients.

Parameter	Normotensive patients	Cushing’s syndrome	*P* value
n	10	8	
Sex(M/F)	5/5	1/7	NS
Age(year)	47.20 ± 15.80	40.13 ± 13.64	NS
Body mass index(kg/m^2^)	22.48 ± 2.74	26.96 ± 4.30	0.016
Systolic blood pressure(mmHg)	123.33 ± 12.86	136.63 ± 14.29	NS
Diastolic blood pressure(mmHg)	74.6 ± 10.41	92.88 ± 9.57	0.001
Fasting glucose(mmol/L)	5.06 ± 0.49	5.96 ± 1.79	NS
White blood cell count (10^9^/L)	7.53 ± 2.66	10.15 ± 2.38	0.045
Creatinine (µmol/L)	95.7 ± 64.25	63.88 ± 6.89	NS
Alanine transaminase (U/L)	20.8 ± 8.19	32.63 ± 21.39	NS
Glutamic-oxal(o)acetic transaminase (U/L)	22.1 ± 12.32	23 ± 12.52	NS
Morning plasma cortisol levels (µg/dl)		24.09 ± 5.68	
(normal value, 4.3–22.4)			
Midnight plasma cortisol levels (µg/dl)		23.37 ± 3.79	
(normal value, 3.9–16.66)			

All data are shown as mean ± Standard Deviation. NS, Not significant.

### Immunohistochemistry and Masson’s staining

Immunohistochemical stains of TNF-α (Abcam, ab6671), FN (Abcam, ab23751), COLI (Abcam, ab6308) and CD68 (Abcam, ab955) was performed on paraffin-embedded specimens of peri-N from CPA and NT patients as described previously [10]. In addition, peri-N paraffin sections were also performed with Masson’s stain as described before [10].

### Cell culture

Mouse 3T3-L1 preadipocytes were purchased from the type culture collection of the Chinese Academy of Sciences. Mouse brown preadipocytes were established as previously described [11,12]. Cells were isolated from the SVF of peri-N and sub-Q from CPA patients as described previously [13]. Human peri-N and sub-Q were obtained from a 26-year-old female with benign CPA who undertook laparoscopic adrenalectomy. She was 156cm in height and weighed 58kg, and she had typical signs of Cushing’s syndrome, such as a moon face, hirsutism, central obesity and purple striae. Biochemical examination revealed a loss of the diurnal circadian rhythm in serum cortisol levels, an increase of 24 hour urinary free cortisol and suppressed ACTH level. Low- and high-dose dexamethasone administration unable to suppress the cortisol level. Computed tomography of adrenal revealed 27mm x 23mm left adrenal nodule. Cells were grown in Dulbecco’s modified Eagle medium (DMEM) supplemented with 10% foetal bovine serum. Preadipocytes were incubated in serum-free DMEM for 12 hours before treatment. Then, the cells were treated with dexamethasone or vehicle (Sigma, D4902) for 24 hours.

### Statistical analysis

The Student’s t test was performed to analyse normally distributed data, and results were expressed as mean ± standard error. Abnormally distributed data were analysed used the Wilcoxon signed-ranks test.

## Results

### Gene expression in peri-N from CPA patients

In an effort to identify the characteristics of peri-N in CPA patients, samples from EH patients (n = 4) and CPA patients (n = 5) were analysed by microarray (Table 1).

**Figure 1.**
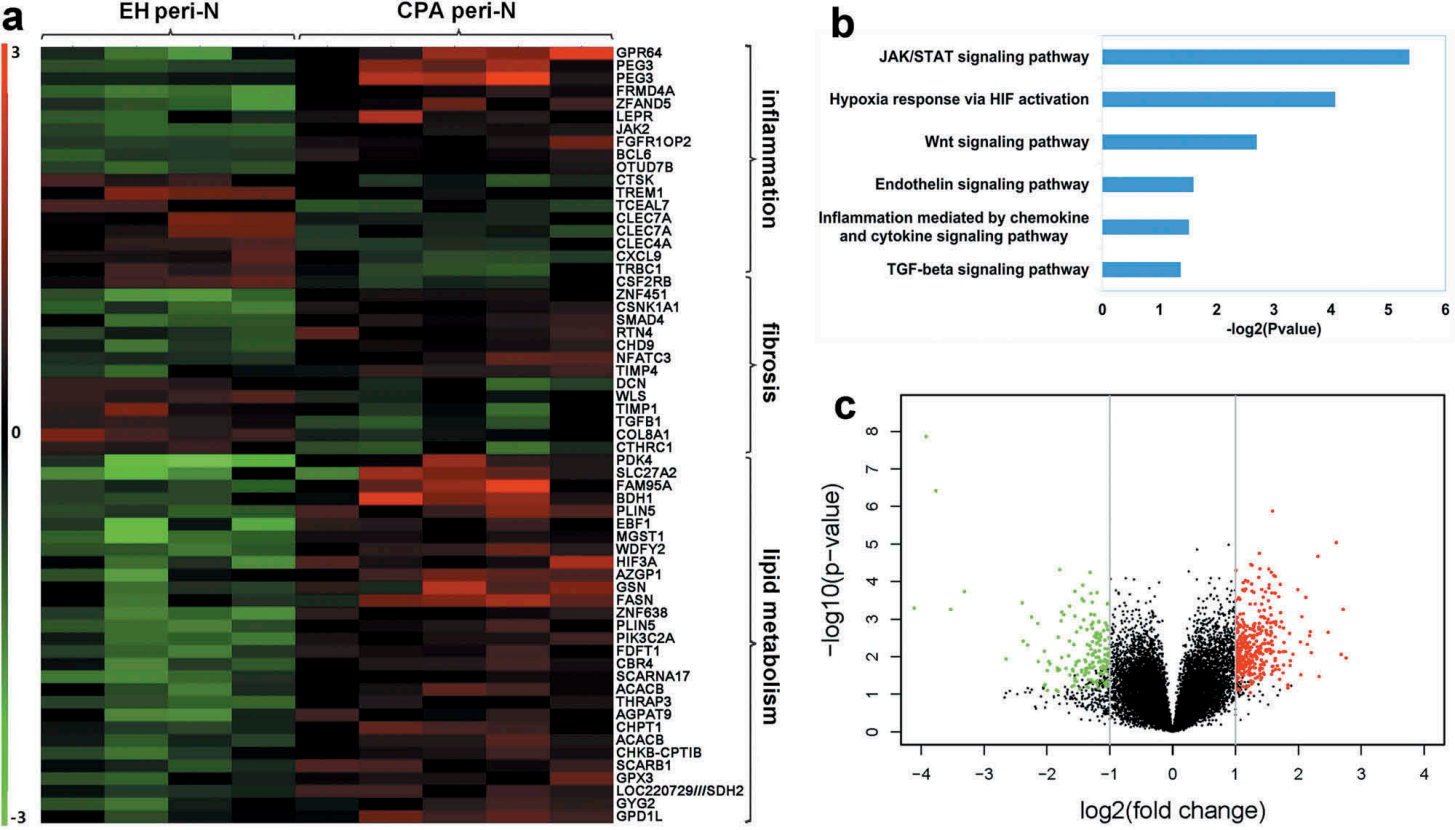
Gene expression profile of peri-N in patients with CPA and EH. (a) Heat map of genes related to lipid metabolism inflammation and fibrosis in peri-N between patients with EH and patients with CPA (fold change > 2.0 or fold change < −0.4; q value<0.05). EH, essential hypertension; CPA, cortisol-producing adenoma; peri-N, perirenal adipose tissue. (b) Pathway analysis showed the most typical pathways in CPA and EH samples. (c) Volcano plot showed differentially expressed genes in peri-N fat from patients with CPA compared with patients with EH. Green symbolizes markedly downregulated genes, and red symbolizes markedly upregulated genes. (fold change ≥2 and p value≤0.05). peri-N, perirenal adipose tissue.

Microarray mRNA expression profiles data revealed dysregulated genes associated with inflammation, fibrosis and lipid metabolism (Figure 1(a)). PANTHER pathway analysis identified signalling mainly associated with inflammation and fibrosis (Figure 1(b)). Volcano plot show differential gene expression profiles in peri-N adipose tissue from patients with CPA compared with patients with EH (Figure 1(c)).

### Clinical characteristics and biochemical measurements in patients with CPA and NT

The characteristics of the patients are described in Table 2. CPA patients had higher white blood cell count, body mass index and diastolic blood pressure than normotensive subjects. Plasma adrenocorticotropic hormone (ACTH) in CPA patients was lower than 1.24 pg/ml (normal value, 7.2–63.3 pg/ml). Urinary free cortisol in CPA patients exceeded 825 µg/24h (normal value, 20.9–292.3 µg/24 h).

### Upregulation of inflammation and fibrosis infiltration in peri-N from patients with CPA

**Figure 2.**
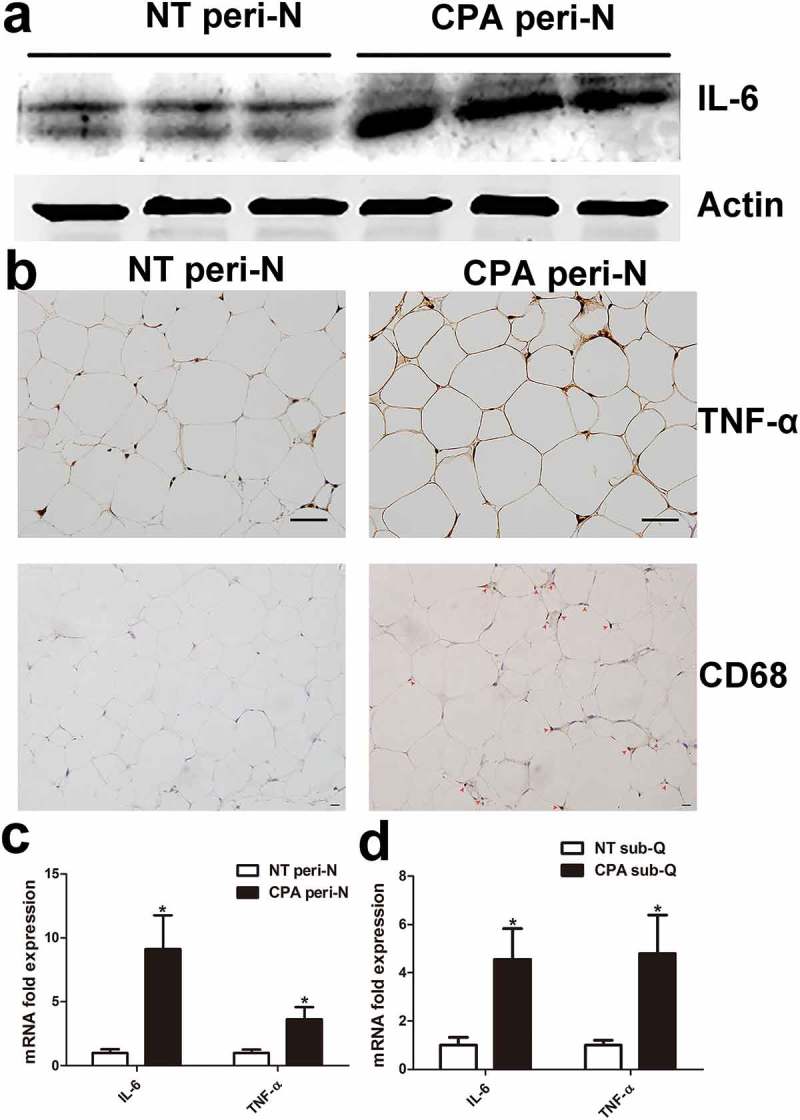
Inflammation change in peri-N from patients with CPA. (a) IL-6 protein expression was detected by Western blot in peri-N from patients with CPA (n = 6) compared with normotension patients(n = 6). (b) TNF-αand CD68 protein expression in peri-N of patients with CPA (n = 5) and controls (n = 5)was assessed by immunohistochemistry (×400). Scale bar, 50 µm. (c, d), IL-6 and TNF-α gene expression in peri-N and sub-Q. NT, normotension, n = 10; CPA, cortisol-producing adenoma, n = 8. sub-Q, subcutaneous adipose tissue. *P < 0.05 vs normotension subjects.

**Figure 3.**
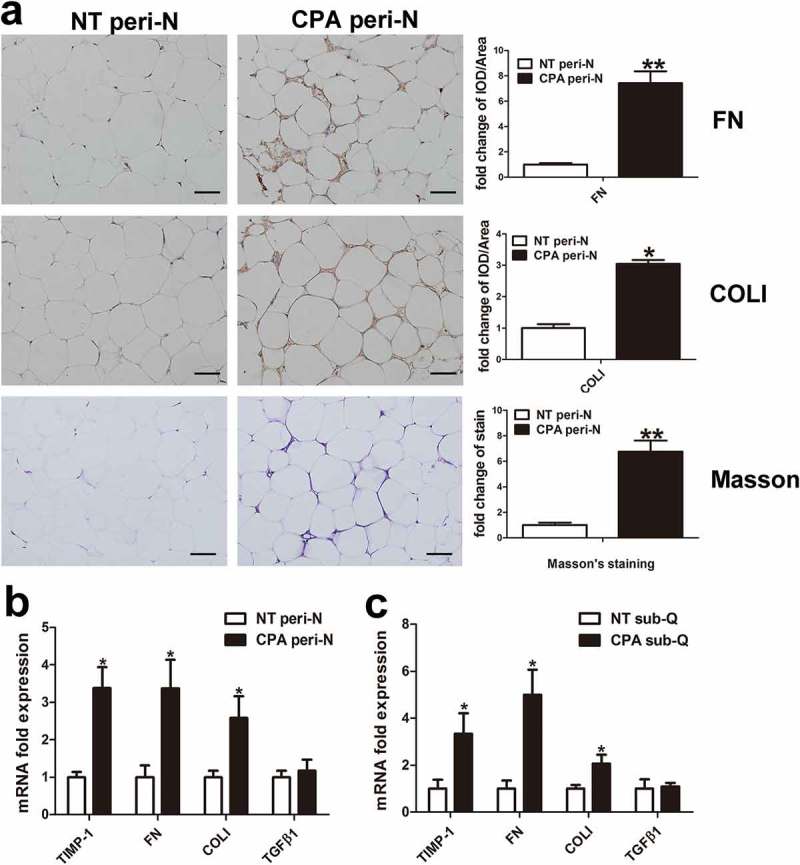
Fibrosis infiltration in peri-N from patients with CPA. (a) Peri-N FN and COLI immunohistochemical and Masson’s staining (×400), NT peri-N, n = 5; CPA peri-N, n = 5. (b) Expression of genes associated with fibrosis in peri-N from CPA group (n = 8) and normotension group (n = 10). Scale bar, 50 µm. NT, normotension; CPA, cortisol-producing adenoma. peri-N, perirenal adipose tissue. (c) Expression of fibrosis genes in subcutaneous adipose tissue from CPA and normotension patients, n = 8. Sub-Q, subcutaneous adipose tissue, n = 10. *P < 0.05 vs normotension subjects.**P < 0.01 vs normotension subjects.

To confirm the microarray data, gene expression was further analysed in the peri-N of CPA patients and controls (NT group). Both mRNA and protein levels of IL-6 and TNF-α were substantially increased in peri-N from CPA patients compared with those in controls (Figure 2(a,b)). The number of macrophage cell specific marker CD68 positive cells observed was notably higher in the peri-N of CPA patients than in the peri-N of controls (Figure 2(b)). The mRNA expression levels of IL-6 and TNF-α was higher in sub-Q of CPA patients than in that of controls (Figure 2(c)). Immunohistochemical analysis demonstrated that FN and COLI protein expression levels were markedly increased in the peri-N of CPA patients compared with those in the peri-N of controls (Figure 3(a)). Accordingly, Masson’s staining also revealed that there was more collagen deposition in the peri-N of CPA patients than in the peri-N of controls (Figure 3(a)). Expression of FN, COLI, tissue inhibitor of metalloproteases 1 (TIMP1) and transforming growth factor-β1 (TGFβ1) mRNA was further detected. The mRNA levels of TIMP1, FN and COLI were markedly increased in the peri-N of CPA patients (Figure 3(b)). In subcutaneous adipose tissue, the levels of fibrosis-related genes including FN, COLI and TIMP1, were remarkably higher in the CPA patients compared with those in the NT patients (Figure 3(c)).

### Increased oxidative stress in peri-N from patients with CPA

**Figure 4.**
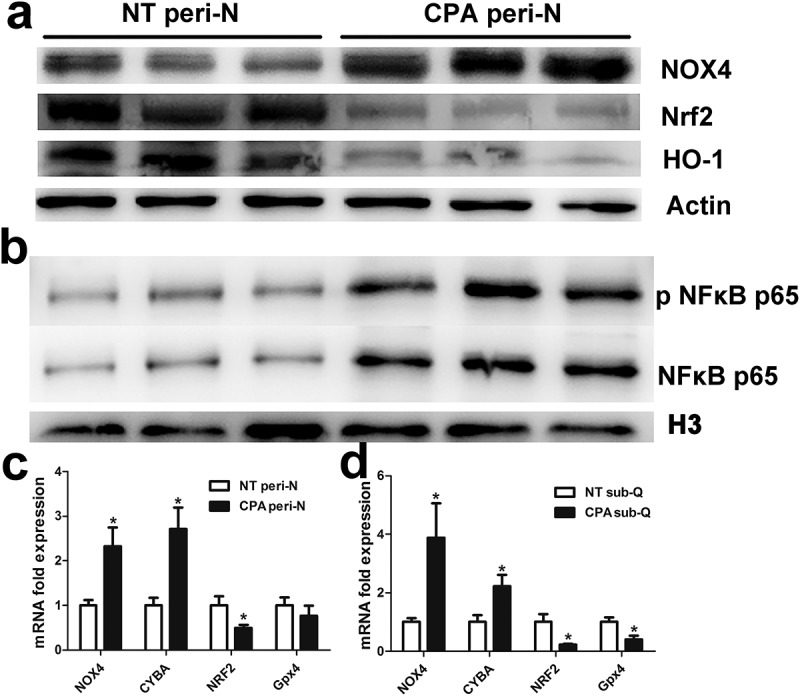
Increased oxidative stress in peri-N from patients with CPA. (a) Western blot analysis of NOX4, Nrf2, HO-1 and β-actin (loading control) protein levels in perirenal adipose tissue; NT peri-N, n = 6; CPA peri-N, n = 6. (b) Western blot analysis of phospho-NF-κB p65 and NF-κB p65 nuclear protein levels in perirenal adipose tissue. H3 was served as a protein loading control. (c, d) Expression changes in reactive oxygen species genes in sub-Q and peri-N from patients with CPA (n = 8) and normotension patients (n = 10). *P < 0.05 vs normotension subjects.

Oxidative stress was analysed in the peri-N of CPA patients. Real time RT-PCR demonstrated that NOX4, glutathione peroxidase 4 (Gpx4) and cytochrome b-245 alpha chain (CYBA) mRNA expression levels were upregulated in both peri-N and subcutaneous adipose tissue from CPA patients compared with controls (Figure 4(c,d)). In contrast, Nrf2 mRNA expression was substantially decreased in both peri-N and subcutaneous adipose tissue in CPA patients (Figure 4(c,d)). Similarly, Western blot showed that NOX4 levels significantly increased, while Nrf2 and HO-1 protein levels were significantly reduced in CPA patients (Figure 4(a)). To explore the role of NF-κB, the nuclear protein levels of phospho-NF-κB and NF-κB in peri-N were analysed by Western blot. As showed in (Figure 4(b)), NF-κB and phospho-NF-κB levels were upregulated in the CPA patients compared with control patients, indicating that NF-κB was activated in the peri-N of CPA patients.

### Effect of dexamethasone on mRNA levels related to adipokines and fibrosis in 3T3-L1 and brown preadipocytes

**Figure 5.**
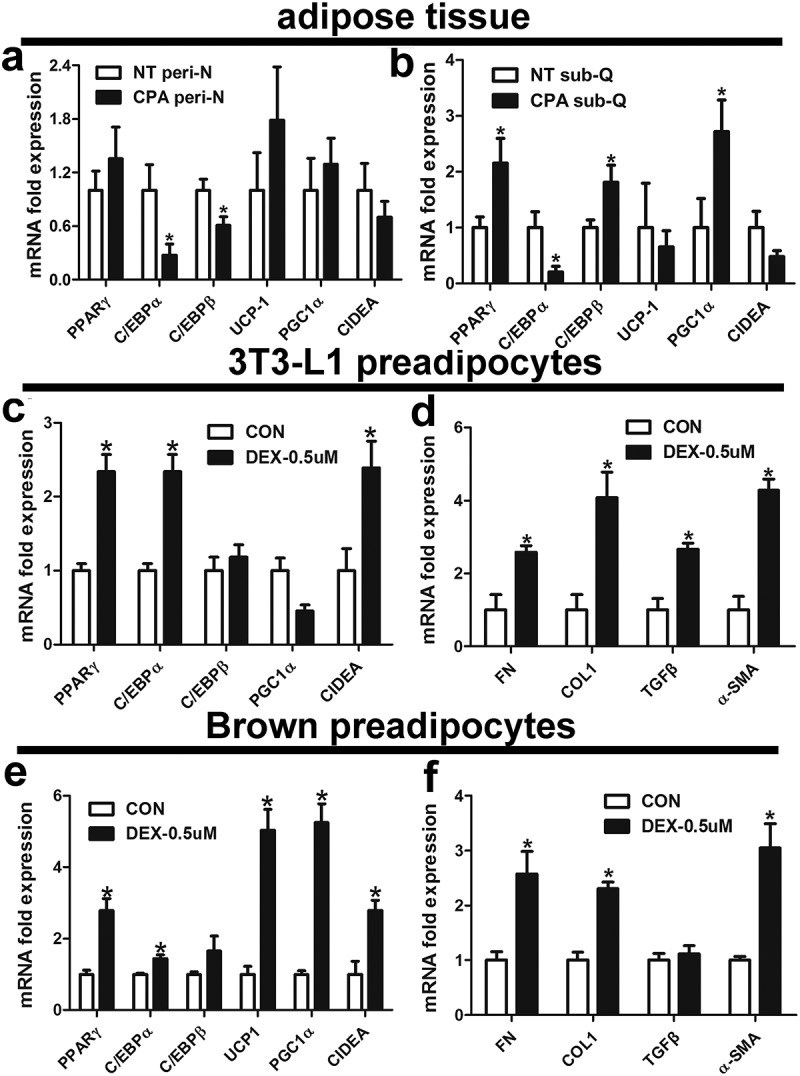
Effect of dexamethasone on mRNA levels related to adipokines and fibrosis in 3T3-L1 and brown preadipocytes. Effect of dexamethasone on mRNA levels related to adipokines and fibrosis inIsolation of SVF cells from peri-N and sub-Q from CPA patient and adipocyte culture in vitro(a, b) Expression of adipokine genes in peri-N and sub-Q from different patient groups. NT, normotension; CPA, cortisol-producing adenoma. *P < 0.05 vs normotension subjects. (c-f) Effect of dexamethasone (DEX) on mouse 3T3-L1 preadipocytes and brown preadipocytes. *P < 0.05 vs CON (control).

To further investigate changes in the function of adipose tissue in CPA patients, we evaluated adipokine markers in peri-N and subcutaneous adipose tissue. Compared with NT group, CCAAT/enhancer binding protein alpha (C/EBPα) and CCAAT/enhancer binding protein beta (C/EBPβ) mRNA levels in peri-N was significantly decreased in the CPA group (Figure 5(a)), whereas peroxisome proliferator activated receptor gamma (PPARγ), C/EBPα and PPARγ coactivator 1 alpha (PGC1α) mRNA levels was remarkably increased in sub-Q of CPA group (Figure 5(b)). To investigate the effect of dexamethasone on adipocytes, mouse 3T3-L1 preadipocytes and brown preadipocytes were treated with dexamethasone. As showed in Figure 5(c,f), dexamethasone significantly increased PPARγ, CIDEA and C/EBPα mRNA levels in 3T3-L1 preadipocytes and brown preadipocytes. Higher UCP1 and PGC1α mRNA expression levels were observed after dexamethasone treatment in brown preadipocytes than in controls (Figure 5(e)). Moreover, dexamethasone induced increase mRNA levels of genes related to fibrosis, including alpha-smooth muscle actin (α-SMA), FN and COLI, in 3T3-L1 and brown preadipocytes (Figure 5(d,f)).

### Isolation of SVF cells from peri-N and sub-Q from CPA patient and adipocyte culture in vitro

**Figure 6.**
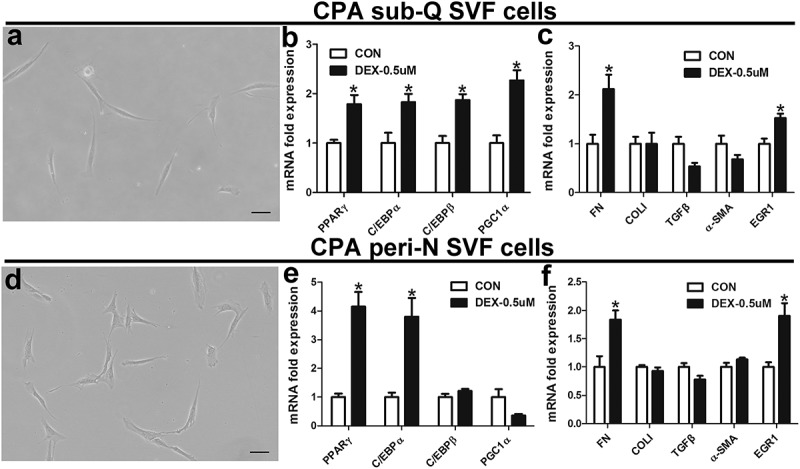
Effect of dexamethasone on SVF cells. (a) and perirenal (d) fat (X100). Effects of dexamethasone on the expression of genes related to fibrosis and adipokines in human perirenal adipose tissue (b, c) and subcutaneous adipose tissue (e, f) and predifferentiated SVF cells. Predifferentiated cells were treated with dexamethasone (DEX, 0.5µM) for 24 hours. *P < 0.05 vs CON (control). Scale bar, 200 µm.

To determine the effect of dexamethasone on SVF cell characteristics and functions, primary preadipocytes were isolated from one CPA patient’s paired subcutaneous and peri-N (Figure 6(a,d)). Next, predifferentiated perirenal and subcutaneous fat SVF cells were treated with dexamethasone (0.5 µM) for 24 hours to examine the mRNA levels of genes associated with adipokines and fibrosis. Dexamethasone remarkably increased PPARγ, PGC1α, C/EBPα and C/EBPβ mRNA expression in predifferentiated CPA sub-Q SVF cells and induced a remarkable increase in FN and early growth response factor-1 (EGR1) mRNA expression (Figure 6(b,c)). Dexamethasone significantly increased PPARγ, C/EBPα, FN and EGR1 mRNA levels in peri-N SVF cells from this CPA patient (Figure 6(e,f)).

## Discussion

In this study, we explored peri-N changes induced by chronic endogenous hypercortisolism due to CPA. We found that oxidative stress, inflammation and fibrosis are notably changed in response to chronic hypercortisolism exposure in patients with CPA, and these features may be involved in adverse metabolic effects.

Adipose tissue fibrosis and inflammation exert an equally important impact on cardiovascular disease [14,15]. A previous study [16] showed that dexamethasone inhibited high fat diet (HFD)-fed mice epididymal adipose tissue macrophages accumulation. The authors also reported [16] that dexamethasone inhibited macrophage recruitment in mature 3T3-L1 cells. The plasma IL-6 level of patients with Cushing’s syndrome was increased compared with normal controls [17].Researchers have found that, despite long-term cure, plasma inflammation markers, including TNF alpha-receptor 1 and IL-6, were higher in Cushing’s syndrome patients than in matched healthy controls [18].Although much is known that acute corticosteroid therapy exerts anti-inflammatory effects [19], little is known about local adipose tissue changes in patients with CPA. Recent research has reported an increased adipose tissue macrophage presence in active Cushing’s disease patients [4]. These findings indicated that chronic exposure to endogenous glucocorticoids may exacerbate inflammation. Irit et al. [20]reported RNA sequencing of subcutaneous fat from patients with Cushing’s disease and non-functioning pituitary adenomas as controls, and the results showed down-regulated expression of transcripts involved in immune function in the Cushing’s disease group. In the current study, our results showed that IL-6 and TNF-α levels were remarkably higher in peri-N from patients with CPA compared with those in patients with NT, which indicated increased inflammation in peri-N from patients with CPA. In addition, in accordance with a previous study [4,21], CD68 positive cells observed was increased in peri-N from patients with CPA compared with that in peri-N from patients with NT.

Henegar et al. [22] found dysregulation of many extracellular matrix components in sub-Q from obese subjects compared with lean subjects. Adipose tissue fibrosis is associated with adipose tissue dysfunction [23]. Our results demonstrated that fibrosis in the peri-N of CPA patients was significantly increased, and genes related to fibrosis were also increased in the sub-Q of CPA patients.

Compared with EH and normotension patients, subcutaneous adipose tissue oxidative stress was increased in Cushing’s syndrome patients [24]. Houstis and co-workers reported that protein carbonyl levels were elevated 110% in dexamethasone treated adipocytes [25].A pervious study also showed persistent metabolic abnormalities during long-term remission Cushing’s syndrome patients [26]. Another study found that oxidative stress as measured by plasma 15-F2t – isoprostane levels was remarkably higher in patients with Cushing’s syndrome than in healthy subjects [27]. Our results revealed that the expression of NOX4 protein was significantly increased, while expression of Nrf2 and HO1 proteins was notably decreased in peri-N from patients with CPA. We also found NF-κB activation in the peri-N of CPA patients. NF-κB plays an important role in inflammatory processes associated with oxidative stress [28]. In the current study, we observed perirenal and subcutaneous adipose tissue dysfunction in CPA patients, which indicated that endogenous hypercortisolism may play an important role in local adipose tissue dysfunction.

In conclusion, our study demonstrated that changes in oxidative stress, inflammation and fibrosis occur in the peri-N of CPA patients. Peri-N dysfunction associated with chronic exposure to hypercortisolism may be involved in metabolic disorders in CPA patients.
